# What are citizens' concerns in urban regeneration? Exploring critical factors for participatory intention, a case from Shanghai

**DOI:** 10.3389/fpsyg.2025.1668925

**Published:** 2025-10-29

**Authors:** Yuan Li, Qin Wei, Jianhao Guo, Yanran Song, Xingyao Wu

**Affiliations:** Shanghai Academy of Fine Arts, Shanghai University, Shanghai, China

**Keywords:** urban regeneration, participatory intention, theory of planned behavior, place attachment, Shanghai

## Abstract

Urban regeneration is a global concern and a crucial component of China's recent urbanization efforts. Among the several stakeholders, citizens' participatory intention in the urban regeneration is closely related to a city's sustainable development. Given China's top-down governance tradition, encouraging public participation has been a challenge for the government. To understand what citizens really care in urban regeneration, this study employed a research model based on theories of planned behavior, place attachment, and place memory, to examine citizens' behavior, focusing on how different variables influenced their participation. The Nanjing West Road Historic and Cultural Area (NWRHCA) in Shanghai has been selected as a case study. Data were collected from 1,137 valid questionnaires, and structural equation modeling (SEM) was applied to test 10 hypotheses regarding the effect paths from six variables to behavioral intention and performance. Additionally, we conducted in-depth interviews to further explain the SEM analysis results. The findings indicate that: (1) place attachment is an important factor in shaping citizens' intention to participate; (2) there are significant negative correlations between attitude and behavioral intention, as well as between memorable urban experiences and behavioral intention; (3) conflicts among stakeholders impede public participation in urban regeneration and (4) willingness of citizens' participation can be enhanced through a “step-by-step” procedural approach, the institutionalization of regeneration process, and the cultivation of residents' place attachment. These findings provided new insights into the conventional approach to urban regeneration in China, exploring citizens' core interest, and highlighting important implications for facilitating diverse forms of public participation in future urban regeneration.

## 1 Introduction

Urban regeneration has been a classic theme in the process of globalization and urbanization across the world (Lang et al., 2016). Since World War II, for a long time the urban regeneration practices have focused on physical rebuilding and economic growth but neglected social issues (Li et al., 2020). However, with tremendous urbanization and social transformations, many countries have paid attention to cultural, social, economic, and environmental powers in urban regeneration (Lang et al., 2016), and treated it as a panacea to solve urban problems (Ercan, 2011). In 1965, Davidoff proposed the term “advocacy planning,” which referred to encouraging citizens' participation in decision-making process of urban development to protect their democratic rights and interests (Davidoff, 1965). The concept of urban regeneration emphasizes the collaboration among government, private sectors, and the community to address regeneration challenges from cultural, social, economic, and environmental perspectives, while enhancing living conditions and preserving the community's historical resources (Biondi et al., 2020; Jones, 2003; Tallon, 2013). Nowadays, public participation has been an essential part of urban regeneration practices (Westerink et al., 2017; Hong, 2018; Lin et al., 2022). By cultivating collective ownership and reinforcing community identity, participatory engagement enhances social cohesion within neighborhoods while ensuring the inclusivity and equity of regeneration initiatives (Hong, 2018; Sun et al., 2025). The cooperation and participation of various stakeholders can generate positive social-cultural and economic impacts in society (Jung et al., 2015).

China's urban regeneration started from the renovation of urban shantytowns and urban villages, which were always featured with large-scale demolition and reconstruction (Lai et al., 2021). During this process, attention to heritage preservation and social network maintenance has promoted the urban regeneration toward a more rational way. Although the urban regeneration practices were traditionally dominated by the state (Wu, 2016), more studies in China have addressed the importance of multiple parties' participation in recent years (Liu et al., 2024; Chen et al., 2023; Xiao et al., 2023; Tang et al., 2022; Gu et al., 2022; Li et al., 2020). In the process of urban regeneration, public participation can help cultivate social capital and promote the community sustainable development (Zhang et al., 2024a). Among various stakeholders, local governments in China have more clout than their Western counterparts, by forming alliances with real estate developers in redevelopment (Li et al., 2020). Citizens seem to assume a minor role in the decision-making process of urban regeneration, but it is they who actually live or work in the renewal areas, and bear the possible environmental loss after regeneration (Zhang et al., 2022). Therefore, citizens' satisfaction should be the final goal of urban regeneration in China, and it is key to pay more attention to this group's participatory intentions and behaviors. In recent decades, several urban regeneration projects have been implemented in Chinese cities. Household-based strategy adopted in the Nanjing's Xiaoxihu project allowed residents to negotiate the pace and scope of renovation. Developers and state-owned enterprises, as seen in the Yongqingfang project in Guangzhou, have engaged in government-led frameworks that incorporated community feedback at various stages. In some cases, grassroots actors have initiated bottom-up participation; for example, the resident-driven renewal efforts along Xinhua Road in Shanghai eventually gained institutional support and evolved into long-term co-creation platforms.

The urban regeneration in Shanghai has been ongoing since the founding of new China. However, it was not until 2015 that this effort was formally defined as “urban renewal” in the official document “Shanghai Urban Renewal Implementation Measures” (Zhou et al., 2019). In the “Master Plan of Shanghai City 2017–2035,” the requirement of “negative growth” was introduced, emphasizing urban regeneration's pivotal role in future urban planning: “We will adhere to a policy of negative growth in the total scale of planned construction land.” Under the goal of negative growth, newly planned construction land will be strictly controlled, making it essential for urban development to rely more on the regeneration of existing developed land. Meanwhile, Shanghai is a forerunner in the practices of historic and cultural area (Lishi Wenhua Fengmaoqu) preservation. As early as 2003, a total of 12 historic and cultural areas in central city were officially designated, including the Nanjing West Road Historic and Cultural Area (NWRHCA). This area features a diverse range of public spaces and residential buildings with varying architectural styles and types, and it used to be home to many celebrities (Chen and Ruan, 2008). In the context of Shanghai nowadays, it is important to integrate historic and cultural area conservation and urban regeneration into local development process.

Current research on citizens' participation in urban regeneration is extensive. Regarding the factors that influence citizens' participatory intention, scholars have paid attention to the institutional limitations, financial burdens, and empowerment processes, but ignoring the various concerns of citizens as witness and stakeholders in urban regeneration. Specifically, the influence of place attachment on citizens' participatory intention in urban regeneration is surprisingly limited. This study aims to bridge this gap. In doing so, our study focuses on residents and working populations in NWRHCA, and examines their behavioral intention toward participation in urban regeneration. Grounded on the well-established theory of planned behavior (TPB), we incorporated place attachment and place memory into the theoretical model to improve its analytical capability. We seek to address the following questions:

(1) What are the critical factors influencing citizens' participatory intention in the urban regeneration of the NWRHCA?
(2) How do these critical factors affect citizens' participatory intentions and behaviors in the decision-making process?
(3) What are citizens' concerns in the process of urban generation?
(4) How can these concerns be addressed to enhance citizens' willingness to participate in urban regeneration?

The remainder of this article will be organized as follows. Section 2 reviews the literature on citizens' participation in urban regeneration, theory of planned behavior, theory of place attachment and place memory, identifying research gaps. Section 3 constructs the research model and develops 10 research hypotheses. Section 4 presents the methodology including an introduction to the study area, questionnaire design, data collection methods, structural equation modeling, demographic profile, and in-depth interviews. In Section 5 we analyze the results of model testing, and examine the associations between citizens' behavioral intentions to participate in the regeneration of NWRHCA and factors we proposed in research hypothesis. Drawing on the findings, Section 6 highlights the theoretical contributions and offers a detailed discussion. Finally in the last section, we summarize our main findings, answer the research questions, and provide accommodations for policy-making in urban regeneration.

## 2 Literature review

### 2.1 Citizens' participation in urban regeneration

In urban development, municipal governments, business groups, and communities all play key roles. In the Chinese context, urban regeneration has been predominantly the tasks of governments and state-owned enterprises (Liu et al., 2024). Only in the renewal of old communities, residents' participation is given priorities because the projects have direct relation with residents' life (Xiao et al., 2023; Li et al., 2024; Sun et al., 2025). In other types of urban regeneration such as preserved regeneration of historic districts, citizens' concerns seem to be ignored. Based on Arnstein's “ladder of citizen participation,” Li et al. (2020) proposed three levels of citizen participation achieved in the process of urban regeneration, including non-participation, tokenism, and citizens empowerment.

There are several factors that might affect citizens' participation in urban regeneration. In community renewal, empowerment has a direct positive relationship with public participation awareness, and impacts residents' participatory willingness through community identity and trust relationship (Sun et al., 2025). For effective resident participation in neighborhood rehabilitation, government incentives, financial incentives, information sharing and communication, and trust among stakeholders play critical roles (Xiao et al., 2023; Li et al., 2020, 2024). An investigation for different stakeholders have indicated consensus about what matters in public participation of urban renewal. For instance, both government officials and citizens agree that “timely responses to public inquires” was crucial for successful management of public participation in urban renewal (Liu et al., 2018).

### 2.2 Theory of planned behavior (TPB)

The theory of planned behavior (TPB) proposed by Ajzen (1985) is one of the most influential conceptual frameworks in the field of human action studies. This theory identifies three predictors that affect individuals' behavioral decision-making: (1) attitude toward behavior (AB), referring to the favorable or unfavorable evaluation of the behavior; (2) subjective norm (SN), referring to the perception of expectations from relevant others; (3) perceived behavior control (PBC), referring to the perceived ease or difficulty of performing the behavior (Ajzen, 2002). TPB has been broadly applied in various fields to analyze participation willingness and behaviors, including people' intention to participate in environmentally friendly projects (Huang et al., 2022; Hao et al., 2020), adolescents' intent to participate in activities (Taylor et al., 2012; Ito and Igano, 2022), travelers' willingness to visit specific places (Woosnam et al., 2022; Zhang et al., 2024b; Juan et al., 2020), and the public's intention to participate in urban renewal. Lin et al. (2022) surveyed hundreds of people in Taiwan and discovered that people's perceived benefits in urban renewal projects significantly affected their intention to participate in urban renewal. A study in China employed the modified TPB and demonstrated that enterprise participation intention in old community renewal has been significantly impacted by their attitude (Liu et al., 2024). For residents, the study based on the extended TPB showed that the cognition, attitude, and intention of participation in the old community renewal significantly affected their behaviors (Gu et al., 2022). In a more complex theoretical construct combining the TPB and the expectation-confirmation model, a study about neighborhood micro-renewal in Shanghai indicated the positive relations between residents' participation intent and subjective norms, behavioral attitudes, perceived behavioral control, and perceived usefulness (Tang et al., 2022).

### 2.3 Place attachment and place memory

Place attachment has been theorized in numerous forms since its original inception in the 1970s (Proshansky, 1978; Scannell and Gifford, 2010a). It is a term originated from environmental psychology, referring to emotional, cognitive, or functional bonds between individuals and their meaningful environments, and it also contains a component of the behavioral intention (Giuliani, 2003; Kyle et al., 2004; Lee et al., 2012; Scannell and Gifford, 2013). Most studies supported a model of place attachment consisting of place identity and place dependence (Lewicka, 2011; Anton and Lawrence, 2014; Boley et al., 2021). People with a strong sense of place attachment have been reported to be more inclined to invest their time and effort on the place they feel attached to Stedman (2003). In which, place identity reflects people's similarities between self and place, and their self-definition drawing from the physical environments (Scannell and Gifford, 2010b); and place dependence means that the place is able to maintain a person's activities and lifestyle, or to facilitate the goal achievement (Jorgensen and Stedman, 2001).

Although several studies have indicated that citizens with high place attachment might not devote their time to participate in community affairs (Perkins and Long, 2002; Palmer et al., 2010), most studies have demonstrated a positive relation between place attachment and public engagement in community and urban affairs (Anton and Lawrence, 2016; Hesari et al., 2020; Shin and Yang, 2022; Zhu, 2022). For residents, when they feel a strong bond between themselves and their neighborhood, and sense that memories are formed, they would be led to the formation of participatory processes to maintain their living environment (Scannell and Gifford, 2010b; Wu et al., 2019; Hesari et al., 2020). For employees working in certain urban area, related studies are limited. Despite spending the majority of their lives in these areas, it appears they have been largely overlooked in civic affairs. Scholars have paid more attention to residents, developers, authority officials, professionals, or even tourists (Chen et al., 2023; Li et al., 2020; Liu et al., 2024).

In the process of urban regeneration, citizens' place attachment is influenced by the change of the place. Ellenbogen and Trivic (2024) proposed a dynamic (re)production of place attachment, which implied that residents' active and meaningful participation in the regeneration project was essential to the promotion of place attachment. Place attachment might be damaged by removal of social spaces, resident relocation, and changing populations in urban regeneration (Lomas et al., 2021). The change of urban environment can have a positive influence on residents' place attachment if they perceive the changes as upgrading (Von Wirth et al., 2016). In some cases, residents' place attachment can be strengthened in the pre-relocation stage of regeneration due to the improved self-esteem evoked by the announcement (Pan and Cobbinah, 2023). In addition to the impact of urban regeneration on the place attachment, people's attitudes toward urban regeneration can also be affected by the place attachment. A study in Shaoguan, China has indicated that strong place attachment is able to significantly improve residents' support intentions for urban regeneration (Xu et al., 2022). Some scholars believed that citizen participation was a key component of place attachment, and held great potential for effective regeneration outcomes (Falanga, 2022). However, place attachment could also be one of the factors that hinder local minorities accepting government-led urban regeneration plan and pushed them fighting for their rights of participation (Zhai and Ng, 2013).

Place memory is the predictor of place attachment by linking people to events and concepts that may have happened in the place (Ratcliffe and Korpela, 2016; Li and Zhao, 2021), and it is relevant with the perception of urban heritage (Falanga, 2022). Places remember through physical features such as monuments, architectural style of their buildings, inscriptions on walls, etc. (Lewicka, 2008). Urban heritages carry the collective memory of daily life and place identity, and the urban regeneration may change not only physical fabrics, but also emotional attachment among local people (Chen X. et al., 2020). In addition to collective memory, people's autobiographical memory also constructs their attachment to the place. The autobiographical memory of an individual contains his or her own life experiences or the personal events experienced in a specific context at a certain time (Robinson, 1986; Brewer, 1986). A place encapsulates and recalls the memory of experiences and events there (Li and Zhao, 2021). And the memory of special experience in a place may play a conscious or unconscious guiding role in the future decision making (Pillemer, 2003).

### 2.4 Research gaps

Extant studies have indicated that, (1) several factors can affect people's participatory intentions in urban regeneration; (2) the TPB has been widely adopted as a fundamental model to examine public participation in urban renewal; (3) in most cases, there is a positive relation between place attachment and public participation in urban affairs, as well as between place attachment and people's support for urban regeneration; (4) place memory can serve as a predictor of place attachment. However, scholars have neglected two aspects. First, Existing studies on the role of place attachment in public participation in urban regeneration have overlooked citizens' participation in the context of historic districts in Chinese cities. As noted in the literature review, researchers have paid enough attention to the associations between the place attachment and people's engagement in various community and urban affairs, as well as how the urban regeneration has interacted with residents' place attachment. However, in a historic preserved district, the place attachment of residents and working populations is closely related with the local cultural heritages and historic buildings, which might influence citizens' intentions to participate in the regeneration of these artifacts. Therefore, it is reasonable to hypothesize that place attachment has a direct impact on citizens' participatory intentions in urban regeneration. Second, to our knowledge there are few studies that link people's place memory to their intentions to participate in urban regeneration practices. However, both the memories living in and the events experienced in a place might affect citizens' future decisions in urban affairs. Therefore, we identified the place memory in the NWRHCA as “memorable urban experiences” (MUEs) and hypothesize it as a predictor of place attachment and participatory intention in urban regeneration.

## 3 Hypothesis development and research model

As a classic theoretical framework for behavioral analysis, the TPB has been widely employed by scholars to examine individual intentions and behaviors. In this study, TPB serves as the basis for analyzing citizens' participatory intentions and behaviors in urban regeneration. Building on this framework, we also incorporate insights from the literature on place attachment, which has been shown to positively influence public participation in community and urban affairs. Specifically, we integrate the constructs of place identity and place dependence into our theoretical model. Furthermore, owing to place memory as a significant predictor of place attachment, it has been additionally introduced into the model in the form of MUEs. Citizens in the NWRHCA have both the rational logic of ordinary individuals—whose decisions are influenced by their own attitudes, the opinions of those around them, and their perceived behavioral control—and emotional connections shaped by the area's historically valuable scenes and collective memories. These emotional bonds, in turn, affect their willingness to participate in urban regeneration. Therefore, in developing the research hypotheses, this study integrates the variables from the TPB, place attachment, and place memory to examine their combined influence on participatory behavior.

### 3.1 Hypothesis development

**Figure 1:**
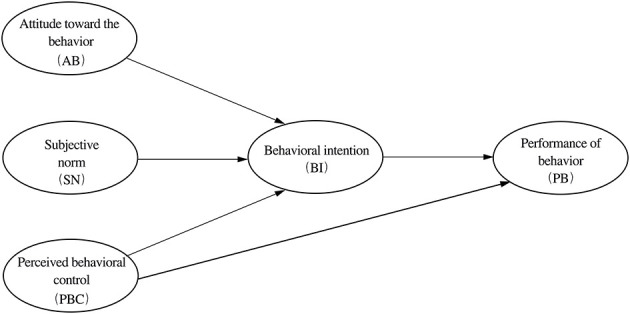
TPB model framework. Source: Ajzen (1985).

Based on the theory of planned behavior (Figure 1), attitude toward behavior (AB) indicates someone's evaluation to the given behavior as favorable or unfavorable. In this study, attitude points to the assessment to the regeneration of the NWRHCA of a citizen who lives or works here. It is reasonable and acceptable that those who have a optimistic attitude toward urban regeneration are more willing to participate in this practice. And several previous studies have also proven it (Liu et al., 2024; Tang et al., 2022; Gu et al., 2022). Therefore, the following hypothesis is proposed:

**H1**. AB significantly and positively affects behavioral intention (BI).

Subjective norms (SN) denote the perceived pressure from others regarding an individuals' potential participation behavior. In the regeneration practice of NWRHCA, citizens' intentions to participate might be influenced by their neighbors, friends, colleagues, employers, and residential committees. Given the government's predominant role in urban renewal, residents may not believe their opinions are decisive but feel that the expectations of significant people around them are influential (Tang et al., 2022). Furthermore, collectivism is advocated by Chinese culture (Ru et al., 2019), which shapes decision-making processes in social affairs. Based on the analysis above, we propose the second hypothesis:

**H2**. SN significantly and positively affects BI.

Perceived behavioral control (PBC) refers to people's perception of ease or difficulty of performing the behavior. It is the predictor of not only behavioral intention but also the final performance of behavior (Tang et al., 2022). Prior studies have verified that as individuals become more positive about PBC, their intention to perform behavior strengthens (Hao et al., 2020; Neto et al., 2020; Shi et al., 2021). In some cases, the behavioral action was also positively affected by individual's ability to engage in social affairs (Meng et al., 2019). Based on the previous studies, two hypotheses are put forward:

**H3**. PBC significantly and positively affects BI.
**H4**. PBC significantly and positively affects PB.

Generally we believe that the actual performance of behavior (PB) is the consequence of behavioral intention. Only when people are willing to engage in affairs, they would perform behaviors, and this causal relation has been verified in many studies (Neto et al., 2020; Gu et al., 2022; Liu et al., 2024). Therefore, in this study we postulate the following:

**H5**. BI significantly and positively affects PB.

People who live or work in a historic preserved district perceive the value of urban built environment, and gain unforgettable memories from previous experiences. The memorable tourism experiences (MTEs) is one of the core concepts explored by scholars, which denotes the experiences that tourists can actively recall at the end of the tourism (Zhang et al., 2018). Studies in tourism indicated that memorable tourism experiences could create positive memories, which enhanced tourists' strong attachment to attractions (Loureiro, 2014), and then further influenced their behavioral intentions (Tsai, 2016). There are studies that have developed several measurement scales for MTEs, which then have been verified by different researchers (Wei et al., 2019). For tourists, an unforgettable experience is determined by not only destination related attributes but also their psychological factors. These factors include hedonism, novelty, involvement, social interaction, serendipity, meaningfulness, refreshment, local culture, knowledge, and adverse feelings (Wei et al., 2019; Tsai, 2016; Kim, 2014). In this study, we proposed that citizens' memorable urban experiences (MUEs) in a specific urban area is similar with tourists' memorable experiences in a certain destination. And three hypotheses about the relations between MUEs and PI, PD, and BI are posited:

**H6**. MUEs significantly and positively affects PI.
**H7**. MUEs significantly and positively affects PD.
**H8**. MUEs significantly and positively affects BI.

Places become personally important through people's memories, cultural significance, and physical comfort (Scannell and Gifford, 2013). The person-place bonds would be strengthened when place is incorporated into one's self-definition or guarantees individuals' goal achievement. Extant studies show that place attachment is an important predictor of the behavioral intentions in future decision making such as revisiting a destination, engaging in climate change, and word-of-mouth communications of a place (Tsai, 2016; Scannell and Gifford, 2013; Jeong et al., 2019). Accordingly, we propose the last two hypotheses:

**H9**. PI significantly and positively affects BI.
**H10**. PD significantly and positively affects BI.

### 3.2 Theoretical model of this study

**Figure 2:**
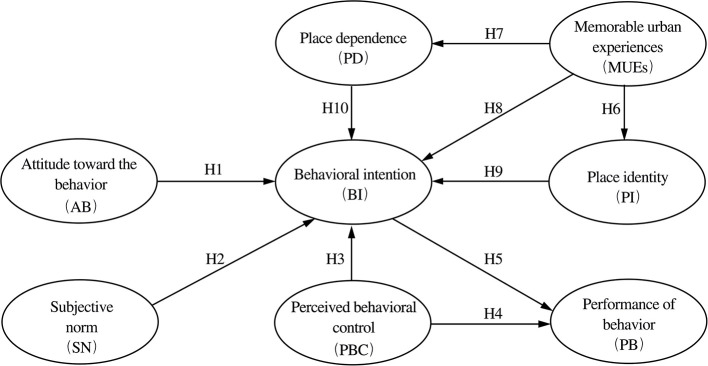
Theoretical model of this study.

In this study we employed the TPB model framework to elucidate the effect of AB, SN, and PBC on citizens' behavioral intentions and actions in the participation of regeneration in the NWRHCA. A historic preserved district encompasses a lot of old buildings and cultural heritages, which have been demonstrated to be able to produce memorable places in the city (Feliú et al., 2020). We adapted the term of MTEs from tourism studies to create the concept of MUEs, and then established the causal relations between MUEs, PI, PD, and BI. By integrating TPB, MUEs, and place attachment, this study aims to identify key determinants that affect people's participatory willingness and the outcomes in urban regeneration practices. This approach extends the original TPB model, enhancing its explanatory power. We try to explore a comprehensive theoretical framework applicable to analyzing citizens' participation in the urban regeneration, as illustrated in the research model in Figure 2.

## 4 Methodology

### 4.1 Study area

**Figure 3:**
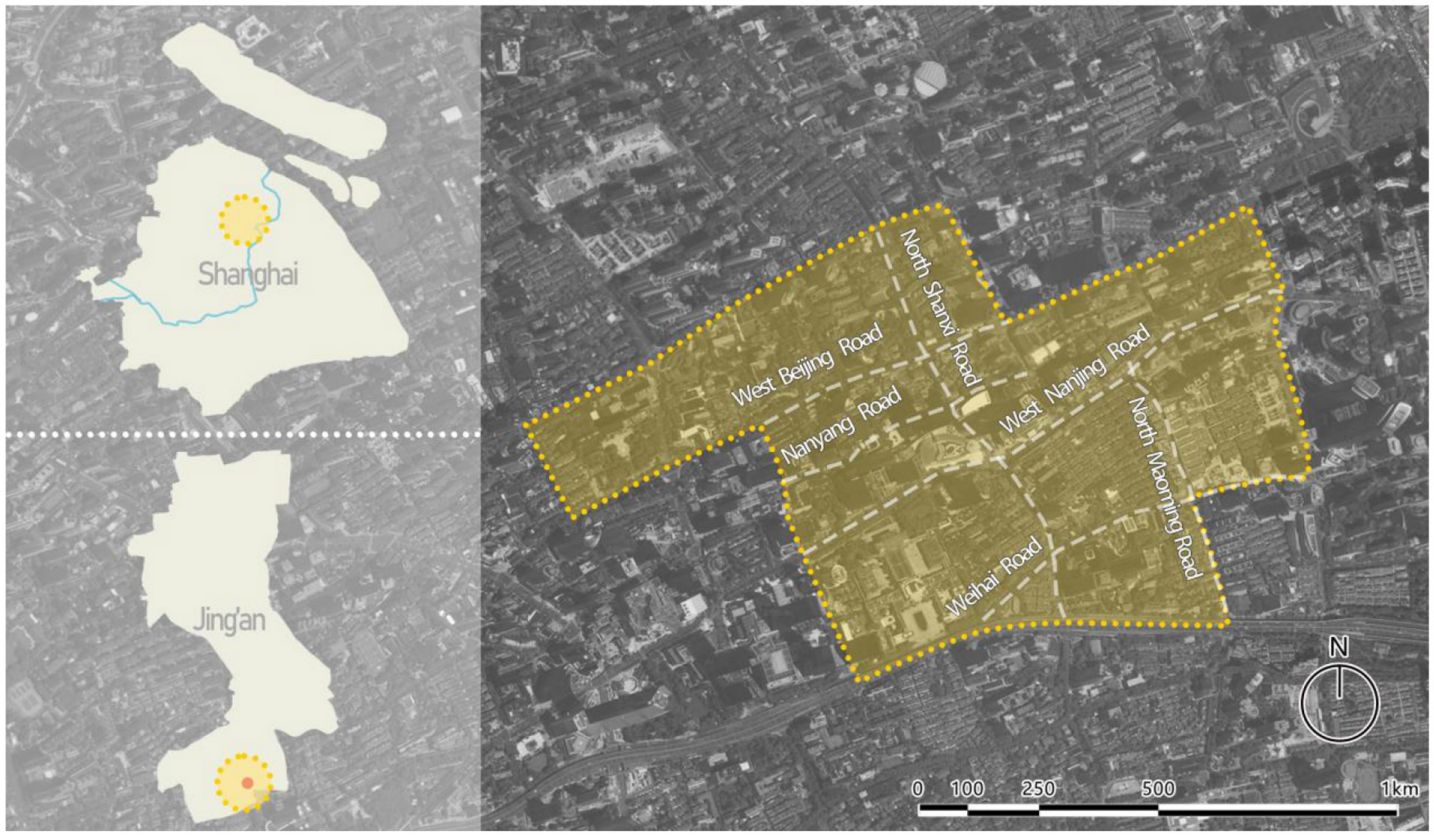
Study area.

**Figure 4:**
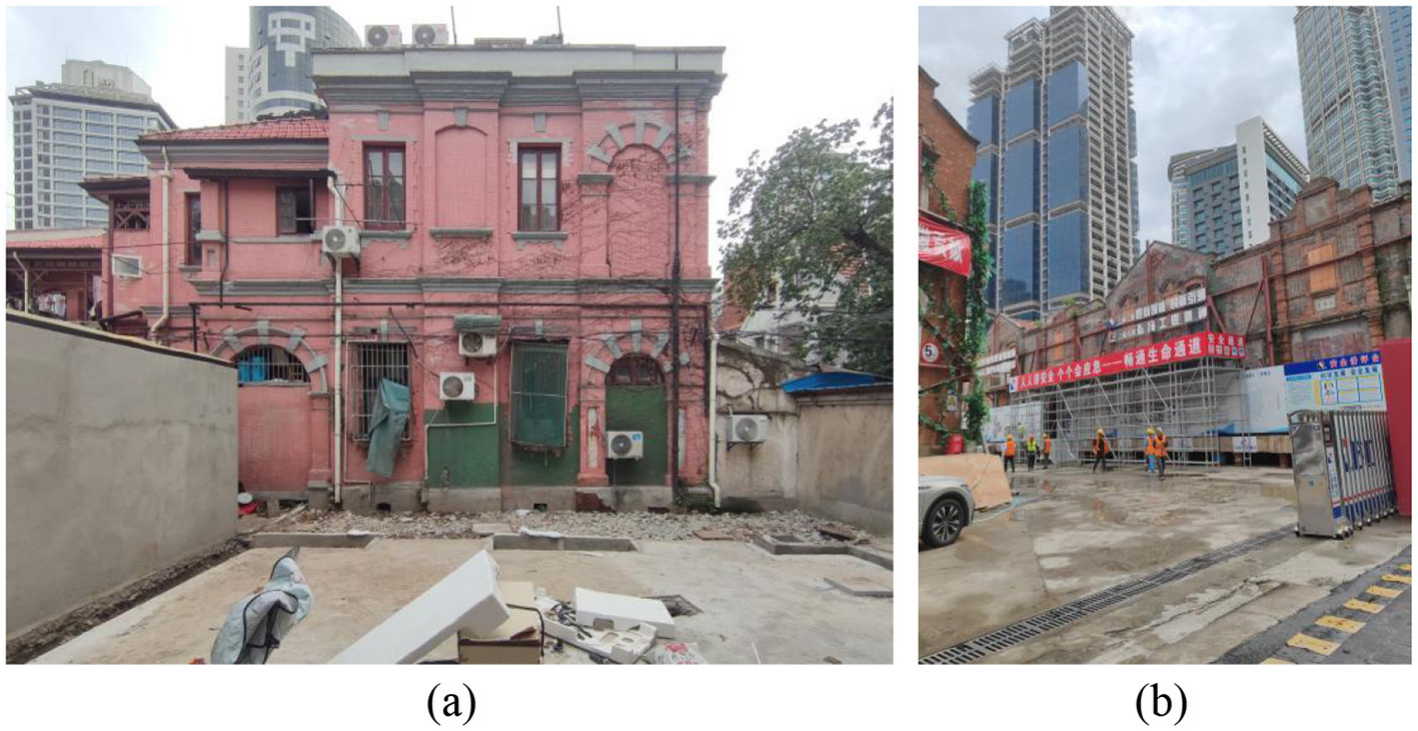
Urban scenes in the NWRHCA. **(a)** The exterior facade of an old house. **(b)** A Shikumen building in Zhangyuan.

The NWRHCA is one of 12 historic and cultural areas in central Shanghai. Located in the southern part of Jing'an District, NWRHCA spans 1.38 square kilometers (Figure 3). By 2024, the district was home to approximately 30,000 permanent residents. Additionally, nearly 100,000 populations, primarily between the ages of 25 and 45, work in this area. NWRHCA contains a variety of historical buildings that reflect the development of Shanghai's cultural soft power, including the Shikumen architecture, Garden Houses, and other twentieth-century architectural heritages. More importantly, NWRHCA is a model for urban regeneration. Through the revitalization and restoration of its historic buildings, the district has not only introduced new cultural and commercial elements but also enhanced the area's vitality and improved the quality of life for its residents. Figure 4 presents urban scenes from the NWRHCA, captured by authors during fieldwork in the study area. This set of images was taken in two field projects of regeneration in the NWRHCA case. Figure 4a shows the renovation of the exterior facade of an old house in the intersection of Beijing West Road and Shannxi South Road; Figure 4b depicts a Shikumen building in Zhangyuan that has been transformed into a cultural and commercial space.

### 4.2 Questionnaire design

In this study we used a questionnaire method to collect data. The questionnaire contains three sections. The first explains background the geographic scope of Nanjing West Road historic preserved district, the second contains the basic information of respondents, and the third is the questions to evaluate the latent variables in the research model. The questionnaire was designed using a five-point Likert scale, with answer options ranging from strongly disagree (1) to strongly agree (5). The scale items for variables were constructed from the previous literature, and Table 1. lists the details of measurement questions as well as the references. In the measurement of MUEs, we modified the scales of MTEs into four variables that closely related with urban life rather than tourism experiences. We performed expert interviews on the questionnaire draft and revised it. And then a pre-survey was conducted by undergraduate students face to face, and the questionnaire was further improved considering both respondents' reactions and an initial reliability test to create the final version.

**Table 1:** Measurement items of variables.

**Variables**	**Items**	**Source**
**Attitude toward behavior (AB)**		
AB1	It is necessary to perform the PR-NWRHCA.	Ma et al., 2018 Si et al., 2020 Tang et al., 2022
AB2	It is beneficial for my life to perform the PR-NWRHCA.	
AB3	It is enjoyable for me to see the PR-NWRHCA.	
AB4	The advantages of protective regeneration overweigh disadvantages.	
**Subjective norms (SN)**		
SN1	Most people around me support the PR-NWRHCA.	Hao et al., 2020 Chen J. et al., 2020 Tang et al., 2022 Xiao et al., 2023
SN2	People who are important to me think I should support the PR-NWRHCA.	
SN3	Community/residential committee/work unit expects me to participate in the PR-NWRHCA.	
SN4	I usually take advice from people who are important to me.	
**Perceived behavior control (PBC)**		
PBC1	If I want, I am confident that I can participate in the PR-NWRHCA.	Lin et al., 2022 Poliakoff and Webb, 2007 Tu et al., 2020
PBC2	I have enough time and energy to participate in the PR-NWRHCA.	
PBC3	I have the ability to participate in the PR-NWRHCA.	
**Memorable urban experiences (MUEs)**		
MUEs1	I feel happy living in the NWRHCA.	Wei et al., 2019 Chen and Rahman, 2018
MUEs2	I have good social relationship here in the NWRHCA.	
MUEs3	The life here in the NWRHCA is meaningful.	
MUEs4	I can remember the appearances of important buildings and streets here in the NWRHCA.	
**Place dependence (PD)**		
PD1	For the urban areas I like, the working/living environment in the NWRHCA is the best.	Kyle et al., 2004 Lee et al., 2012
PD2	I could not imagine any working/living environment better than the NWRHCA.	
PD3	I enjoy working/living in the NWRHCA more than any other places.	
**Place identity (PI)**		
PI1	I feel the NWRHCA is a part of my life.	Jorgensen and Stedman, 2006
PI2	I identify strongly with the NWRHCA.	
PI3	I feel that I can really be myself in the NWRHCA.	
**Behavioral intention (BI)**		
BI1	I am willing to participate in the PR-NWRHCA.	Liu et al., 2024 Ru et al., 2019 Xu et al., 2020
BI2	I will try to participate in the PR-NWRHCA.	
BI3	I would like to continue to participate in the PR-NWRHCA.	
**Performance of behavior (PB)**		
PB1	I have the working conditions to participate in the PR-NWRHCA.	Xu et al., 2020 Liu et al., 2024 Tonglet et al., 2004
PB2	I have tried to participate in the PR-NWRHCA.	
PB3	I have been participating in the protective regeneration in NWRHCA.	

PR-NWRHCA means protective regeneration of Nanjing West Road Historic and Cultural Area.

### 4.3 Data collection

The sample data were collected randomly from the NWRHCA based on a two-wave survey in October, 2024. The two-wave survey consists of a pre-survey and a formal survey. In the pre-survey, questionnaires were distributed by undergraduate students to residents or working populations face to face. Every question can be explained in detail to the respondents, and the reactions about expressions of questions can be collected. We received 78 responses in total in this wave and 68 responses were valid. We revised some details in expression to create the final questionnaire. In the formal survey, most questionnaires were distributed online and then 1,176 responses returned, in which 1,069 were valid. All the question input and data collection were conducted by the largest questionnaire survey website in China, Questionnaire Star. The survey link was forward to the WeChat chat room of residents under the assistant of the residents' committees, and some were completed face to face in the fieldwork. All online questionnaires were distributed in WeChat chat rooms composed of the target population, and both IP filtering and screening questions on the Questionnaire Star were employed to ensure the accuracy of respondent eligibility.

### 4.4 Structural equation modeling (SEM)

In this study structural Equation Modeling (SEM) was used as the primary method to test the research hypotheses. SEM is a confirmatory statistical approach in which causal hypothesis must be based on a specific theoretical framework. We developed our 10 hypotheses based on the theories of planned behavior, place attachment, place memory, and memorable tourism experiences. Therefore, SEM would be an appropriate method for analyzing citizens' participatory intentions in urban regeneration. Through SEM, we evaluated the measurement quality and examined the relationships among eight latent variables. In the comparison of different groups (working populations vs. residents), we applied multi-group analysis (MGA) in SEM.

Referring to the study of Shin and Yang (2022), we selected four demographic characteristics that may affect place attachment and behavior of participating in urban regeneration including age, education, period of residence in Shanghai, and household income as control variables.

### 4.5 Demographic profile

The questionnaire respondents' socioeconomic characteristics have been summarized in Table 2. Among the 1,137 respondents, residents and working populations account for 17.33% and 41.60%, and the remaining 41.07% both live and work in the NWRHCA. There are 722 men and 415 women, with the majority (68.87%) aged from 18 to 35 years old. The educational attainment of the respondents is relatively high, more than three-fourths of whom have the bachelor degree or higher. Citizens here mainly work in government, professional firms, or retail settings such as stores and shopping malls. Regarding the period of residence in Shanghai, nearly 38% of the respondents have lived in Shanghai for 4–10 years. In addition, household incomes distribution follows an “olive” shape, with a small proportion earning less than CNY 5,000 or more than CNY 30,000, while most fall between CNY 5,001 and 30,000.

**Table 2:** Demographic characteristics of questionnaire respondents (*N* = 1,137).

**Items**	**Demographic**	**Frequency**	**Percentage**
Type	Employee	473	41.60
	Resident	197	17.33
	Employee & resident	467	41.07
Gender	Male	722	63.50
	Female	415	36.50
Age	Under 18	24	2.11
	18–35	783	68.87
	36–59	303	26.65
	60 and over	27	2.37
Education	Junior high and below	26	2.29
	Senior high	60	5.28
	Junior college	177	15.57
	Bachelor	700	61.57
	Master or higher	174	15.30
Occupation	Government agencies and institutions	183	16.09
	Professional and technical work	273	24.01
	Administrative work	237	20.84
	Commercial services	227	19.96
	Agriculture, forestry, animal husbandry, fishery, and water conservancy	51	4.49
	Production or transportation	78	6.86
	Serviceman	6	0.53
	Student	29	2.55
	Freelancer	12	1.06
	Others	41	3.61
Period of residence in Shanghai	Within 1 year	38	3.34
	1–3 years	213	18.73
	4–10 years	431	37.91
	11–20 years	187	16.45
	More than 20 years	268	23.57
Household income/month (CNY, yuan)	Less than 5,000	39	3.43
	5,001–10,000	253	22.25
	10,001–20,000	461	40.55
	20,001–30,000	229	20.14
	More than 30,000	155	13.63

### 4.6 In-depth interviews

In order to gain a deeper understanding of the SEM analysis results, in-depth semi-structured interviews were conducted. These interviews aimed to explore explanations for SEM model results that differed from findings in previous studies. Specifically, the questions were designed to examine citizens' memories in the NWRHCA and the influence of these memories on the behavioral intention to participate in the regeneration practices. In addition, the relation between attitude and behavioral intention was also investigated. To ensure diverse information sources, we selected interviewees with different socioeconomic backgrounds. Seven individuals participated in the interviews, and their information is shown in Table 3. The interview included five questions, and allowed participants to express their views freely in a non-restrictive manner. These questions involved interviewees' memories in the NWRHCA, their attitude and participatory intention regarding regeneration, the drivers behind their decisions, and their expectations for the future urban regeneration. Each interview lasted about 20 min and was recorded. The data were then analyzed to help explain the SEM findings.

**Table 3:** Information of interviewees.

**Code**	**Title**	**Age**	**Residence period in Shanghai**	**Occupation**
C1	Ms. M	60 and over	More than 20 years	Retired
C2	Mr. Anonymous	60 and over	More than 20 years	Retired
C3	Mr. H	60 and over	More than 20 years	Retired
C4	Ms. P	18–35	4–10 years	Government worker
C5	Mr. Anonymous	36–59	More than 20 years	Worker
C6	Ms. T	18–35	More than 20 years	Planner
C7	Mr. G	36–59	11–20 years	Commercial services

## 5 Results of model testing

### 5.1 Reliability and validity of measurement items

Before analysis, the reliability and validity of the data were assessed using the online tool SPSSAU (https://spssau.com/?107000000). The Cronbach's α coefficient indicated the internal consistency of the scale, and higher values represent higher reliability. A coefficient of 0.7 or higher is generally considered acceptable. Validity assessment included both convergent and discriminant validity. Convergent validity, which reflects the degree of relevance of questions within the same dimension, was assessed by factor loading (FL), combined reliability (CR) and average extracted variance (AVE). It is acceptable that the FL is above 0.6, CR value is above 0.7 and AVE value is above 0.5. According to Fornell and Larcker (1981), discriminant validity measures the degree of difference between different dimensional constructs, which is examined by comparing the square root of the AVE value with the inter-construct correlations. The correlation coefficient between each pair of latent variables should be lower than the square root of the corresponding AVE.

As shown in Table 4, the Cronbach's α coefficient, CR, and AVE have met criteria for reliability and convergent validity. Table 5 further shows that most of the AVE square root values (the bold fonts) meet the required standards, and therefore it can be judged that the structural model design of this study is acceptable (Lin et al., 2022).

**Table 4:** Results of reliability and validity analysis of the final scale.

**Variables**	**FL**	**Mean**	**Cronbach's α**	**CR**	**AVE**
AB	0.695–0.730	4.25	0.806	0.807	0.511
SN	0.711–0.752	4.06	0.823	0.824	0.539
PBC	0.713–0.753	4.11	0.774	0.733	0.532
MUE	0.697–0.723	4.31	0.800	0.801	0.502
PD	0.706–0.738	4.16	0.766	0.766	0.522
PI	0.687–0.721	4.33	0.748	0.751	0.502
BI	0.734–0.761	4.20	0.789	0.789	0.556
PB	0.761–0.788	4.08	0.815	0.816	0.597

**Table 5:** Results of Fornell-Larcker discriminant validity testing.

	**AB**	**SN**	**PBC**	**MUEs**	**PD**	**PI**	**BI**	**PB**
AB	**0.715**							
SN	0.700	**0.734**						
PBC	0.649	0.665	**0.729**					
MUEs	0.608	0.439	0.611	**0.709**				
PD	0.607	0.655	0.667	0.644	**0.722**			
PI	0.554	0.408	0.586	0.722	0.617	**0.708**		
BI	0.589	0.612	0.682	0.618	0.694	0.624	**0.745**	
PB	0.503	0.568	0.679	0.544	0.646	0.606	0.726	**0.773**

The bold values are AVE square root.

In the groups analysis (working populations vs. residents), we defined respondents who work in the NWRHCA as working populations (473 cases), and those who live, or both live and work, in this area as residents (664 cases). The CFA results show that both the reliability and validity of working population sample are good (Cronbach's α ≥ 0.754, FL ≥ 0.710, CR ≥ 0.764, AVE ≥ 0.520). For the resident sample, the values of Cronbach's α (≥0.726), FL (≥0.624), and CR (≥0.725) are good, but the AVE values range from 0.412 to 0.551. Given that the FL and CR values meet the required thresholds, the AVE values are close to 0.5, and the questionnaire items are adapted from established scales with all questions traceable to the existing literature, we conclude that the reliability and validity of the resident sample are also acceptable.

### 5.2 Goodness-of-fit of the models

We analyzed the goodness-of-fit of the measurement and structural models using AMOS 26.0. In both models, most fit indices were at a good level, and all indices met the acceptable standards (Table 6). These results suggested that each latent construct was accurately measured, and the structural model effectively revealed the relations among variables.

**Table 6:** Fit indices of the model in this study.

**Indices**	**Level of acceptance fit**	**Measurement model**	**Structural model**
χ^2^/*df*	< 5.0 acceptable; < 3.0 good	2.561	3.062
GFI	>0.8 acceptable; >0.9 good	0.953	0.938
AGFI	>0.8 acceptable; >0.9 good	0.940	0.920
RMSEA	< 0.1 acceptable; < 0.08 good	0.037	0.043
CFI	>0.9	0.971	0.952
TLI	>0.9	0.966	0.941
IFI	>0.9	0.971	0.952

We also examined the model fit in the multi-group analysis (MGA) to test measurement and structural invariance. Specifically, we compared the unconstrained model with the measurement weights model to assess metric invariance, and the measurement weights model with the structural weights model to evaluate structural invariance. The model comparison results indicate that, after constraining the factor loadings, the model fit indices did not decline significantly (CFI decreased by < 0.001, RMSEA increased by < 0.001). Moreover, Δχ^2^ = 19.246 with Δ*df* = 19 and *p* = 0.441 > 0.05, suggesting that measurement invariance holds. In other words, the measurement scales of all items for the latent variables are consistent across working population groups and resident groups, ensuring the fairness of subsequent group comparisons of path coefficients. After constraining the path coefficients, Δχ^2^ = 47.856 with Δ*df* = 26 and *p* = 0.006 < 0.05, indicating that overall structural invariance does not hold. This suggests that at least one of the paths differs significantly between population groups, requiring further identification of the specific paths exhibiting such differences.

### 5.3 Results of structural model testing

**Figure 5:**
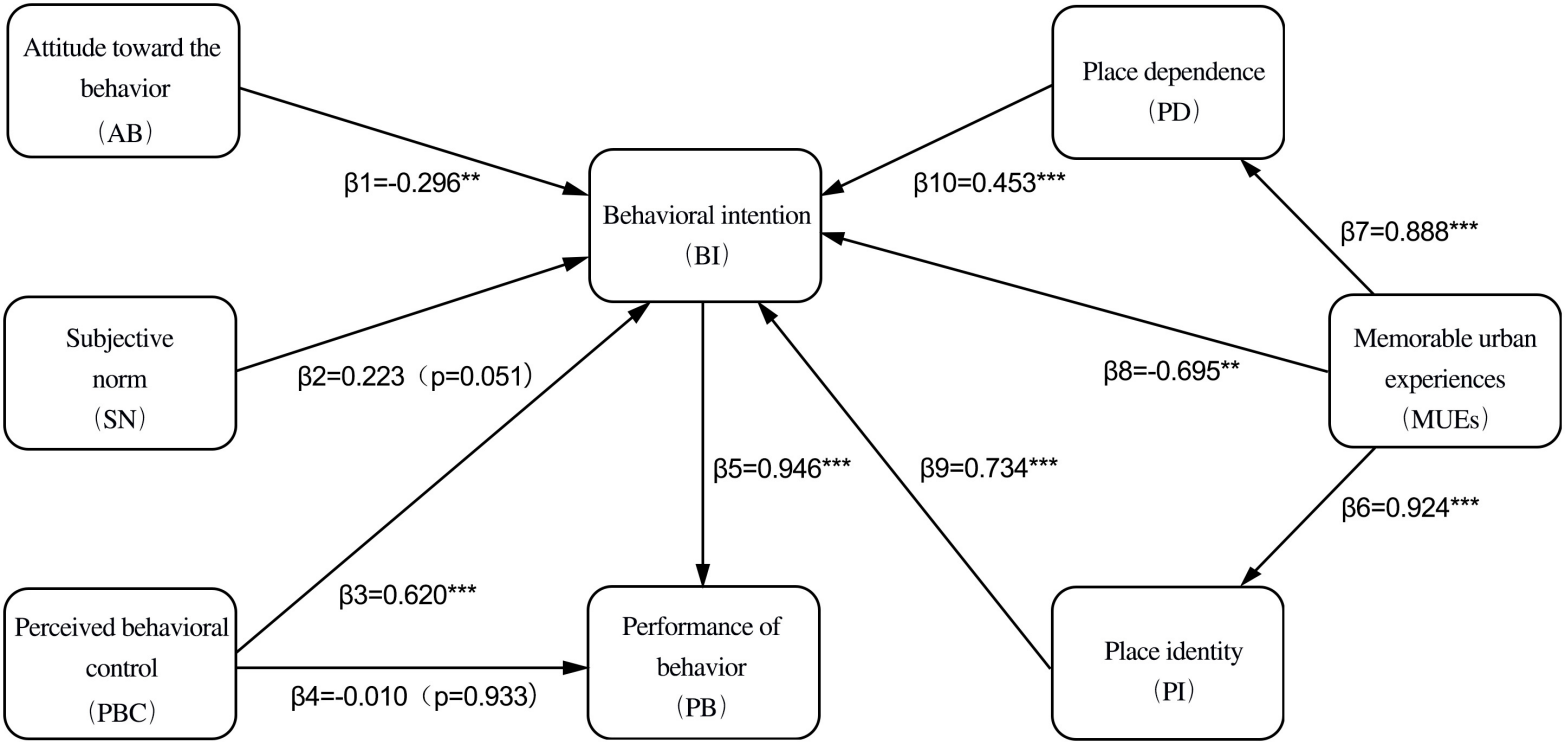
Output of structural equation model. ^*^P < 0.05; ^**^P < 0.01; ^***^P < 0.001.

The Table 7 reveals the relations between four control variables and four dependent variables. After excluding the influence of four control variables, the analysis revealed that four of the 10 proposed paths were not significant, while the remaining six hypotheses were supported (Figure 5, Table 8). The results indicated that behavioral intention had a strong impact on behavioral performance (H5, β = 0.946, *P* < 0.001). The three constructs of the TPB model—AB, SN, and PBC—had varying effects on behavioral intention. Specifically, behavioral intention was negatively influenced by attitude (H1, β = −0.296, *P* < 0.01), positively affected by perceived behavioral control (H3, β = 0.620, *P* < 0.001), and showed no significant relationship with subjective norms (H2, *P* = 0.051). In the part of place attachment, MUEs had a substantial positive effect on place identity (H6, β = 0.924, *P* < 0.001) and place dependence (H7, β = 0.888, *P* < 0.001), but a negative impact on behavioral intention (H8, β = −0.695, *P* < 0.01). In contrast, both place identity and place dependence positively influenced citizens' behavioral intention (H9, β = 0.734, *P* < 0.001; H10, β = 0.453, *P* < 0.001).

**Table 7:** Correlation results of control variables and dependent variables.

**Paths**	**Factor loading**	***P*-value**
Age → PD	−0.031	0.241
Age → PI	0.061^*^	0.016
Age → BI	−0.063^*^	0.036
Age → PB	−0.034	0.168
Education → PD	−0.023	0.412
Education → PI	−0.009	0.728
Education → BI	0.029	0.346
Education → PB	−0.015	0.566
Residence length → PD	0.039	0.160
Residence length → PI	0.046	0.087
Residence length → BI	−0.003	0.922
Residence length → PB	−0.042	0.109
Household income → PD	−0.026	0.344
Household income → PI	−0.038	0.150
Household income → BI	−0.004	0.909
Household income → PB	0.004	0.868

^*^P < 0.05; ^**^P < 0.01; ^***^P < 0.001.

**Table 8:** Hypothesis for the structural models.

**Hypothesis**	**Factor loading**		**Hypothesis Supported**
H1	AB → BI	−0.296^**^	No
H2	SN → BI	0.223(*p* = 0.051)	No
H3	PBC → BI	0.620^***^	Yes
H4	PBC → PB	−0.010(*p* = 0.933)	No
H5	BI → PB	0.946^***^	Yes
H6	MUEs → PI	0.924^***^	Yes
H7	MUEs → PD	0.888^***^	Yes
H8	MUEs → BI	−0.695^**^	No
H9	PI → BI	0.734^***^	Yes
H10	PD → BI	0.453^***^	Yes

^*^P < 0.05; ^**^P < 0.01; ^***^P < 0.001.

In the MGA, as shown in Table 9, the influencing paths for the working population were consistent with those identified in the overall analysis. Moreover, the critical ratios of differences (*Z*-scores) indicated no statistically significant differences (absolute values < 1.96) between the working population and resident samples. However, for the resident sample, the paths from AB → BI, MUEs → BI, and PI → BI were not significant, whereas all of these paths were significant in the working population sample. This outcome appears contradictory to the model comparison results and requires further research.

**Table 9:** The influencing paths and critical ratios for differences.

**Paths**	**Employees (β)**	**Residents (β)**	***Z*-score**
AB → BI	−0.468^**^	−0.146(*p* = 0.370)	1.146
SN → BI	0.162(*p* = 0.425)	0.296(*p* = 0.089)	0.299
PBC → BI	0.885^**^	0.359^*^	−1.729
PBC → PB	0.088(*p* = 0.394)	0.145(*p* = 0.472)	0.206
BI → PB	0.867^***^	0.770^***^	−0.158
MUEs → PI	0.897^***^	0.963^***^	1.490
MUEs → PD	0.957^***^	0.816^***^	−0.038
MUEs → BI	−0.951^**^	0.268(*p* = 0.788)	1.137
PI → BI	0.737^***^	−0.051(*p* = 0.957)	−0.865
PD → BI	0.593^**^	0.348^***^	−1.381

^*^P < 0.05; ^**^P < 0.01; ^***^P < 0.001.

### 5.4 Interview findings

The findings of in-depth interviews deepen our understanding of the SEM analysis results. The interviews reveal a significant disconnect between past urban regeneration practices and the expectations or interests of local residents (Table 10). Specifically, residents are more concerned with issues “inside” their homes, such as termites damaging timber structures, rather than decorations of urban facades. Meanwhile, professionals involved in urban affairs tend to prioritize the implementation of design schemes and policy proposals. However, the interviews also indicate that regeneration practices often involve disproportionately high costs compared to the perceived or actual benefits, raising concerns about their overall effectiveness and efficiency.

**Table 10:** The mismatch between expectations and outcomes.

**Code**	**Viewpoints**
C3	“I propose one suggestion, if (government) plans to decorate buildings in the future, not only the facade, but also the indoor environment should be concerned.”
C1	“Many of the people living here are quite elderly, and because the ceiling height is higher than usual—each floor is 3 m—our third floor is equivalent to the fourth floor in other buildings. Therefore, we strongly desire to install an elevator. Some elderly residents have proposed this, but they were told that, since we live in a preserved building, no changes can be made. There is space in the back alley and in the garden—why can't they consider the needs of residents? A few elderly residents are unable to walk up the stairs, and the younger people have already moved away. While the environment is quite comfortable, as people get older, climbing stairs becomes increasingly difficult.”
C6	“In the urban regeneration of an area such as NWRHCA, the practice has shifted to stock renewal rather than large-scale construction. In this process we must bear a high communication cost, instead of just completing technical tasks. Furthermore, this process is quite long and may ultimately lead to no results.”

Both residents and employees working in NWRHCA in the interviews agreed that a preserved regeneration in this area is necessary and beneficial. However, this generally positive attitude does not necessarily translate into a strong intention to participate in urban regeneration efforts. Citizens with more positive attitude usually hold specific expectations for the regeneration practice and hope that the outcomes will align with their interests. When the expectations are not met, their willingness to participate diminishes significantly, highlighting the importance of aligning regeneration outcomes with public needs and priorities.

The findings also indicate that citizens in the area tend to undervalue their capacity to participate in regeneration, deferring to decisions from the governments rather than asserting their own opinions (Table 11).

**Table 11:** Citizens' perceived lack of responsibility for participation.

**Code**	**Viewpoints**
C5	“I am not professional in these issues.”
C2	“It is none of my business; they (the government) do what they decide.”
C7	“We are not professional, and we need selections offered by the state rather than us proposing strategies.”

Citizens are generally unfamiliar with the processes involved in urban regeneration and lack accessible channels to obtain sufficient information. In other words, they are not decisive actors in the regeneration process. As a result, when asked about the participatory intentions in regeneration practice, many perceive it as beyond their responsibility, citing a lack of relevant knowledge or experience in decision-making. A more positive attitude toward the preserved regeneration may inadvertently lead citizens to overestimate the complexity and technical expertise required, thereby reinforcing a sense of inadequacy and ultimately discouraging their intention to participate.

Additionally, the interviews revealed some difficulties in public participation caused by passive social integration (Table 12). In the global context, the social integration is often promoted by relocating poor households to neighborhoods with less poverty (Li and Feng, 2024). However, in the case of NWRHCA, new residents introduced by urban regeneration projects have led to discomfort among original residents.

**Table 12:** The conflicts between new and original residents.

**Code**	**Viewpoints**
C1	“They quarreled or even fought over the occupation of public balcony.”
C4	“It is hard to bring everyone together to work on regeneration, due to the various conflicts between residents.”

In most cases, original residents possess more memories and personal stories associated with the place. However, urban regeneration projects result in significant changes not only to the physical environment but also to the demographic composition. New residents with entirely different social and cultural backgrounds have been introduced as tenants, workers, or property owners, replacing part of the original populations. For the remained original residents, conflicts are unavoidable. These tensions may foster distrust or resentment toward new neighbors, making residents less willing to engage collaboratively in urban affairs such as the preserved regeneration.

## 6 Discussions

This study integrated theories of planned behavior, place attachment, and place memory to investigate public participation in urban regeneration, examining the factors that influenced citizens' decision-making processes. Our theoretical contributions have three aspects. First, we find that both place attachment and place memory play critical roles in shaping citizens' participatory intention in urban regeneration. While place attachment positively influences participatory intention, place memory unexpectedly exerts a negative effect. Second, attitude and memory—factors that were previously observed positively influenced behavioral intention—has a negative impact on public participation intention in the regeneration of NWRHCA. These counterintuitive results suggest underlying tensions among stakeholders in the Chinese urban regeneration context. Third, we further explore the nature of these conflicts through in-depth interviews. Conflicts arise when practice is not align with residents' expectations, when citizens perceive participation is beyond their responsibility, and when residents with various backgrounds have to live together. The research model we proposed could offer broad applicability for studying public participation in urban regeneration, providing valuable insights for both academic research and policy development.

### 6.1 The critical role of place attachment

This study found that when citizens have memorable experiences in a certain urban area, these memories would positively and significantly influence their attachment to the place, with a strong effect. That is, living and working in NWRHCA enable citizens to create unforgettable memories about urban images and life events, and such positive memories further enhance their dependence on or identification with the area. These empirical results have been observed in several study fields (Xu et al., 2009; Scannell and Gifford, 2013; Tsai, 2016) but rare in urban renewal research. Our findings also illustrated that the intention to participate significantly influences the performance of behavior, while place attachment plays a key role in shaping the intention. From the analysis it is clear that memorable urban experiences significantly strengthen citizens' place attachment, thereby facilitating their intention to participate in urban regeneration. However, abundant memories result in their cherish for urban images and stories, creating a fear of change and disruption that hinders their participatory intention.

Therefore, enhancing people's comprehension with each other while alleviating their worries about the outcomes of regeneration is crucial to fostering public participatory intentions. On the one hand, citizens who are familiar with neighbors and admire the urban space and iconic buildings may develop an emotional connection with the place, which increases their willingness to participate in urban regeneration. On the other hand, if the outcomes of urban regeneration can be guaranteed (usually by the government in China) to be align with citizens' expectations, avoiding disruptions to their daily life, they would have a more positive participatory intention.

### 6.2 The underlying tensions among stakeholders in urban regeneration

It is generally accepted that factors such as attitude and memory have positive impact on behavioral intentions (Liu et al., 2024; Tsai, 2016). However, the negative correlations observed between AB and BI, as well as between MUEs and BI, contradict this general consensus. This suggests that certain underlying tensions in specific context have resulted in these unexpected consequences.

The inconsistency between attitude and behavioral intention is akin to cognitive dissonance, which can exist at various stages in decision-making. For cognitive dissonance to occur, a person must have the desire to achieve a certain outcome and must value that outcome (Juvan and Dolnicar, 2014). In our study, interview findings indicated that when citizens held a positive attitude toward the urban regeneration in NWRHCA, they expected a renewal that is able to improve their living conditions. However, what they have witnessed in past practices is urban facades receiving more attention, rather than residents' living conditions such as structures, interior decorations, or water and electricity pipelines. Despite their admiration for urban regeneration, they were unwilling to participate because “usually it is a work on the surface” (C2, Mr. Anonymous). This is a conflict between the local government and residents.

In some cases citizens felt that they were “not capable” of contributing to urban regeneration. China's urban redevelopment has traditionally been highly state-led. Both central and local state play significant roles in sponsoring and controlling the process of urban regeneration projects (Wu, 2016; Lai et al., 2021). During this process, enterprises can alleviate governments' financial pressure and effectively control the project implementation (Liu et al., 2024). In contrast, citizens lack accessible channels to obtain relevant information or voice their opinions, and they are aware that they are not decisive actors in the execution of projects. This top-down approach has led many people to undervalue their ability to participate in urban regeneration. Some believe that they lack expertise, while others feel it is beyond their responsibility. It has been demonstrated that people will intend to participate in the place change when they feel their actions would influence decision making (Anton and Lawrence, 2016). Our study indicated that citizens with positive attitudes toward regeneration held expectations for ideal outcomes but felt that their intentions and actions would not influence the consequence. Therefore, they were reluctant to participate in the practice. This is a conflict between citizens and government, as well as between citizens and enterprises.

When residents are satisfied with their living environment, it is possible that they have little intention to participate in community renewal that might alter it (Tang et al., 2022). In our study, a similar correlation existed between MUEs and BI. The SEM results showed that citizens with abundant memorable urban experiences in the NWRHCA tended not to participate in urban regeneration initiatives. This resistance stemmed from a fear of change, because place change caused by urban regeneration might erase historic and cultural landmarks, displace old communities, or introduce new people that original residents do not accept. For example, one interviewee C7 (Mr. G) said he disagreed to share space in front of his door with new neighbors or other citizens. If urban regeneration leads original residents' life to an undesirable way, it is understandable that they refuse to participate in the regeneration. This is a conflict among different citizens.

### 6.3 Other findings and implications

The findings indicated that SN did not have significant impact on BI, suggesting that citizens in NWRHCA prioritize their own opinions over attitudes from relevant others when making decisions. Therefore, in this context enhancing external conditions for public participation should not take public opinion influence into consideration. In addition, PBC significantly affected BI, but its impact on PB was insignificant. Citizens are more inclined to participate in urban regeneration when they perceive themselves as capable of doing so. In contrast, low PBC would hinder their intention, which has been discussed above. However, a strong PBC cannot lead to a positive action of participation, as the performance of participatory behavior requires several additional conditions, such as enough funding, satisfying expectations, and encouragements from the state.

## 7 Conclusions

This study revealed some citizens' contradictory perceptions in the process of urban regeneration. It is not a simple binary framework consisting of support or disapproval, willing or unwilling, rather a multifaceted system. Our theoretical model validated strong predictive power and explanatory capability regarding citizens' participatory intentions and behaviors in NWRHCA, reinforcing our understanding of public participation in China's urban regeneration practice. Several main findings have been demonstrated in response to the four research questions.

First, this study examined six critical predictors toward citizens' participatory intentions and behaviors using questionnaire data from 1,137 respondents and interviews with seven participants. The results showed that AB, PBC, MUEs, PI, and PD are critical factors influencing BI, which furthermore strongly predicts PB. Moreover, both PI and PD are significantly influenced by MUEs. Second, the influencing paths from PBC to BI, PI to BI, PD to BI, and BI to PB were all positively significant, while MUEs had positive correlations with PI and PD. However, four of the original 10 hypotheses yielded unexpected outcomes: SN had no significant effect on BI, PBC did not influence PB significantly, while both AB and MUEs exhibited significant but negative effects on BI. Third, insights from in-depth interviews can partly explain these unexpected results. We learn that citizens in NWRHCA do really care in urban regeneration are: (1) achieving regeneration outcomes that align with their demands and interests; (2) having decisive roles in the regeneration process; and (3) minimizing regeneration practices' disruptions to their lives.

Finally, rooted in the empirical analysis and discussion, as well as the experiences in past regeneration practices, we put forward several policy implications to foster public participation in urban regeneration. First, a “step-by-step” regeneration strategy can be adopted in the practice. This approach enables residents to participate regeneration process across multiple stages, including planning, decision-making, and implementation. By involving residents at each stage, the strategy allows for the development of tailored regeneration plans that align with the specific needs and preferences of individual households. This participatory, phased approach can effectively address the mismatch between residents' expectations and outcomes of regeneration projects. Second, it is advisable to institutionalize the participatory initiatives, while closely combined with practices. Establishing a formal participatory platform—led by the local government and supported through collaboration with enterprises and citizens—can ensure that residents' voices are heard both before and after the implementation of regeneration projects. Such a platform provides a structured mechanism for incorporating public input into planning and execution, thereby increasing the likelihood that citizens' ideas and concerns are effectively translated into practice. Third, communities can organize participatory activities such as building micro-gardens within neighborhoods. These activities provide opportunities for both original and new residents to work together to create some common community landscapes, fostering mutual understanding and strengthening social cohesion. By cultivating emotional bonds among residents, such initiatives can enhance place attachment, which in turn is likely to increase their intention to participate in future regeneration efforts.

There are several limitations that can be bridged in future research. As we concluded from this study, citizens' participatory intention in urban regeneration is influenced by numerous factors, the system consisting of which is complex. Thus, more theories or factors such as cognition of urban regeneration can be incorporated into the theoretical model in future studies. Second, the contradiction between the path analysis of resident sample and model comparison suggest a study with more balanced sample sizes and a more detailed questionnaire for residents in the future. In addition, comparative studies using the same theoretical model in other historic and cultural areas of Shanghai or in other Chinese cities are essential. Such research, based on larger and more diverse samples, will provide broader insights into public participation in urban regeneration across different contexts.

## Data Availability

The raw data supporting the conclusions of this article will be made available by the authors, without undue reservation.

## References

[B1] Ajzen I. (1985). “From intentions to actions: a theory of planned behavior,” in Action Control: From Cognition to Behavior, eds. J. Kuhl, and J. Beckmann (Berlin; Heidelberg: Springer), 11–39. 10.1007/978-3-642-69746-3_2

[B2] Ajzen I. (2002). Perceived behavioral control, self-efficacy, locus of control, and the theory of planned behavior. J. Appl. Soc. Psychol. 32, 665–683. 10.1111/j.1559-1816.2002.tb00236.x

[B3] Anton C. E. Lawrence C. (2014). Home is where the heart is: the effect of place of residence on place attachment and community participation. J. Environ. Psychol. 40, 451–461. 10.1016/j.jenvp.2014.10.007

[B4] Anton C. E. Lawrence C. (2016). The relationship between place attachment, the theory of planned behavior and residents' response to place change. J. Environ. Psychol. 47, 145–154. 10.1016/j.jenvp.2016.05.010

[B5] Biondi L. Demartini P. Marchegiani L. Marchiori M. Piber M. (2020). Understanding orchestrated participatory cultural initiatives: mapping the dynamics of governance and participation. Cities 96:102459. 10.1016/j.cities.2019.102459

[B6] Boley B. B. Strzelecka M. Yeager E. P. Ribeiro M. A. Aleshinloye K. D. Woosnam K. M.. (2021). Measuring place attachment with the abbreviated place attachment scale (APAS). J. Environ. Psychol. 74:101577. 10.1016/j.jenvp.2021.101577

[B7] Brewer W. F. (1986). “What is autobiographical memory?” in Autobiographical Memory, ed. D. C. Rubin (Cambridge: Cambridge University Press), 25–49. 10.1017/CBO9780511558313.006

[B8] Chen F. Ruan Y. (2008). A comparative study on conservation planning of historic and cultural areas in Shanghai and the response of conservation planning. Urban Plann. Forum 2, 104–110. [in Chinese]

[B9] Chen H. Rahman I. (2018). Cultural tourism: An analysis of engagement, cultural contact, memorable tourism experience and destination loyalty. *Tour. Manag. Perspect*. 26, 153–163. 10.1016/j.tmp.2017.10.006

[B10] Chen J. Huang J. Huang X. Sun S. Hao Y. Wu H. (2020). How does new environmental law affect public environmental protection activities in China? Evidence from structural equation model analysis on legal cognition. Sci. Total Environ. 714:136558. 10.1016/j.scitotenv.2020.136558 31991275

[B11] Chen W. Ye C. Liu Y. (2023). From the arrival cities to affordable cities in China: seeing through the practices of rural migrants' participation in Guangzhou's urban village regeneration. Habitat Int. 138:102872. 10.1016/j.habitatint.2023.102872

[B12] Chen X. Zhu H. Yuan Z. (2020). Contested memory amidst rapid urban transition: the cultural politics of urban regeneration in Guangzhou, China. Cities 102:102755. 10.1016/j.cities.2020.102755

[B13] Davidoff P. (1965). Advocacy and pluralism in planning. J. Am. Inst. Plann. 31, 331–338. 10.1080/01944366508978187

[B14] Ellenbogen N. R. Trivic Z. (2024). Dynamic place attachment in the context of displacement processes: the socio-ecological model. Cities 148:104862. 10.1016/j.cities.2024.104862

[B15] Ercan M. A. (2011). Challenges and conflicts in achieving sustainable communities in historic neighborhoods of Istanbul. Habitat Int. 35:295e306. 10.1016/j.habitatint.2010.10.001

[B16] Falanga R. (2022). Understanding place attachment through the lens of urban regeneration. Insights from Lisbon. Cities 122:103590. 10.1016/j.cities.2022.103590

[B17] Feliú E. G. Vera J. R. Villalón E. P. Castro J. C. P. (2020). Urban modernization and heritage in the historic centre of Santiago de Chile (1818–1939). Plann. Perspect. 35, 91–113. 10.1080/02665433.2018.1512055

[B18] Fornell C. Larcker D. (1981). Evaluating structural equation models with unobservable variables and measurement error. J. Market. Res. 18, 39–50. 10.1177/002224378101800104

[B19] Giuliani M. V. (2003). “Theory of attachment and place attachment,” in Psychological Theories for Environmental Issues, eds. M. Bonnes, T. Lee, and M. Bonaiuto (Aldershot: Ashgate), 137–170.

[B20] Gu T. Hao E. Ma L. Liu X. Wang L. (2022). Exploring the determinants of residents' behavior towards participating in the sponge-style old community renewal of China: extending the theory of planned behavior. Land 11:1160. 10.3390/land11081160

[B21] Hao Y. Wang Y. Wu Q. Sun S. Wang W. Cui M. (2020). What affects residents' participation in the circular economy for sustainable development? Evidence from China. Sustain. Dev. 28, 1251–1268. 10.1002/sd.2074

[B22] Hesari E. Moosavy S. M. Rohani A. Kivi S. B. Ghafourian M. Pour B. S. S. (2020). Investigation the relationship between place attachment and community participation in residential areas: a structural equation modelling approach. Soc. Indic. Res. 151, 921–941. 10.1007/s11205-020-02408-6

[B23] Hong Y. (2018). Resident participation in urban renewal: focused on Sewoon Renewal Promotion project and Kwun Tong Town Centre project. Front. Architect. Res. 7, 197–210. 10.1016/j.foar.2018.01.001

[B24] Huang H. Wei J. Yang R. (2022). Determinants of consumers' intention to participate in automobile recalls for environmental defects: using an extended theory of planned behavior. J. Environ. Plann. Manage. 66, 2151–2170. 10.1080/09640568.2022.2062566

[B25] Ito H. Igano C. (2022). Why students participate in international fieldwork programs:an exploratory study. J. Geogr. Higher Educ. 46, 560–577. 10.1080/03098265.2021.1947205

[B26] Jeong Y. Kim S,-K Yu J,-G. (2019). Determinants of behavioral intentions in the context of sport tourism with the aim of sustaining sporting destinations. Sustainability 11:3073. 10.3390/su11113073

[B27] Jones P. S. (2003). Urban regeneration's poisoned chalice: is there an impasse in (community) participation-based policy? Urban Stud. 40, 581–601. 10.1080/0042098032000053932

[B28] Jorgensen B. S. Stedman R. C. (2001). Sense of place as an attitude: lakeshore owners attitudes toward their properties. J. Environ. Psychol. 21, 233–248. 10.1006/jevp.2001.0226

[B29] Jorgensen B. S. Stedman R. C. (2006). A comparative analysis of predictors of sense of place dimensions: attachment to, dependence on, and identification with lakeshore properties. J. Environ. Manage. 79, 316–327. 10.1016/j.jenvman.2005.08.003 16288828

[B30] Juan Y. Kang S. K. Lee C. K. Choi Y. Reisinger Y. (2020). Understanding views on war in dark tourism: a mixed-method approach. J. Travel Tourism Market. 37, 823–835. 10.1080/10548408.2020.1835789

[B31] Jung T. H. Lee J. Yap M. H. T. Ineson E. M. (2015). The role of stakeholder collaboration in culture-led urban regeneration: a case study of the Gwangju project, Korea. Cities 44, 29–39. 10.1016/j.cities.2014.12.003

[B32] Juvan E. Dolnicar S. (2014). The attitude–behavior gap in sustainable tourism. Ann. Tourism Res. 48, 76–95. 10.1016/j.annals.2014.05.012

[B33] Kim J.-H. (2014). The antecedents of memorable tourism experiences: the development of a scale to measure the destination attributes associated with memorable experiences. Tourism Manage. 44, 34–45. 10.1016/j.tourman.2014.02.007

[B34] Kyle G. T. Mowen A. J. Tarrant M. (2004). Linking place preferences with place meaning: an examination of the relationship between place motivation and place attachment. J. Environ. Psychol. 24, 439–454. 10.1016/j.jenvp.2004.11.001

[B35] Lai Y. Tang B. Chen X. Zheng X. (2021). Spatial determinants of land redevelopment in the urban renewal processes in Shenzhen, China. Land Use Policy 103:105330. 10.1016/j.landusepol.2021.105330

[B36] Lang W. Chen T. Li X. (2016). A new style of urbanization in China: transformation of urban rural communities. Habitat Int. 55, 1–9. 10.1016/j.habitatint.2015.10.009

[B37] Lee J. Kyle G. Scott D. (2012). The mediating effect of place attachment on the relationship between festival satisfaction and loyalty to the festival hosting destination. J. Travel Res. 51, 754–767. 10.1177/0047287512437859

[B38] Lewicka M. (2008). Place attachment, place identity, and place memory: restoring the forgotten city past. J. Environ. Psychol. 28, 209–231. 10.1016/j.jenvp.2008.02.001

[B39] Lewicka M. (2011). Place attachment: how far have we come in the last 40 years? J. Environ. Psychol. 31, 207–230. 10.1016/j.jenvp.2010.10.001

[B40] Li X. Zhang F. Hui E. C.-m. Lang W. (2020). Collaborative workshop and community participation: a new approach to urban regeneration in China. Cities 102:102743. 10.1016/j.cities.2020.102743

[B41] Li Y. Feng X. (2024). Does the poverty concentrate in Shanghai? – Spatial patterns of social housing and its implications for social equality in Chinese cities. Sustainability 16:2009. 10.3390/su16052009

[B42] Li Y. Tao Y. Qian Q. K. Mlecnik E. Visscher H. J. (2024). Critical factors for effective resident participation in neighborhood rehabilitation in Wuhan, China: from the perspectives of diverse stakeholders. Landsc. Urban Plan. 244:105000. 10.1016/j.landurbplan.2023.105000

[B43] Li Z. Zhao Z. (2021). Reliving past experience: memory and rural tourism destination imageas predictors of place attachment. Asia Pacific J. Tourism Res. 26, 1402–1417. 10.1080/10941665.2021.1985545

[B44] Lin S.-M. Lee H.-Y. Hu H.-L. Chien K.-H. (2022). To join the rebuild or not? An exploration of the factors influencing the public's intention to participate in urban renewal. Sci. Prog. 105, 1–29. 10.1177/00368504221140273 36444484 PMC10450478

[B45] Liu B. Wang X. Xia N. Ni W. (2018). Critical success factors for the management of public participation in urban renewal projects: perspectives from governments and the public in China. *J. Urban Plann*. Dev. 144:04018026. 10.1061/(ASCE)UP.1943-5444.0000467

[B46] Liu G. Huang R. Li K. Shrestha A. Wang H. (2024). Exploring the dilemma of enterprises participating in the old community renewal: perspective of managers. Cities 150:105073. 10.1016/j.cities.2024.105073

[B47] Lomas M. J. Ayodeji E. Brown P. (2021). Experiences of place attachment and mental wellbeing in the context of urban regeneration. Health and Place 70:102604. 10.1016/j.healthplace.2021.102604 34157505

[B48] Loureiro S. M. C. (2014). The role of the rural tourism experience economy in place attachment and behavioral intentions. Int. J. Hospital. Manage. 40, 1–9. 10.1016/j.ijhm.2014.02.010

[B49] Ma J. Hipel K. W. Hanson M. L. Cai X. Liu Y. (2018). An analysis of influencing factors on municipal solid waste source-separated collection behavior in Guilin, China by Using the Theory of Planned behavior. Sustain. Cities Soc. 37, 336–343. 10.1016/j.scs.2017.11.037

[B50] Meng X. Tan X. Wang Y. Wen Z. Tao Y. Qian Y. (2019). Investigation on decision-making mechanism of residents' household solid waste classification and recycling behaviors. Resour. Conserv. Recycl. 140, 224–234. 10.1016/j.resconrec.2018.09.021

[B51] Neto I. L. Matsunaga L. H. Machado C. C. Günther H. Hillesheim D. Pimentel C. E.. (2020). Psychological determinants of walking in a Brazilian sample: an application of the Theory of Planned behavior. Transport. Res. F 73, 391–398. 10.1016/j.trf.2020.07.002

[B52] Palmer N. A. Perkins D. D. Xu Q. (2010). Social capital and community participation among migrant workers in China. J. Community Psychol. 39, 89–105. 10.1002/jcop.20419

[B53] Pan Y. Cobbinah P. B. (2023). Embedding place attachment: residents' lived experiences of urban regeneration in Zhuanghe, China. Habitat Int. 135:102796. 10.1016/j.habitatint.2023.102796

[B54] Perkins D. D. Long D. A. (2002). “Neighborhood sense of community and social capital: a multi-level analysis,” in Psychological Sense of Community: Research, Applications, and Implications, eds. A. Fisher, C. Sonn, and B. Bishop (New York, NY: Plenum), 291–318. 10.1007/978-1-4615-0719-2_15

[B55] Pillemer D. (2003). Directive functions of autobiographical memory: the guiding power of the specific episode. Memory 11, 193–202. 10.1080/741938208 12820831

[B56] Poliakoff E. Webb T. L. (2007). What factors predict scientists' intentions to participate in public engagement of science activities? Sci. Commun. 29, 242–263. 10.1177/1075547007308009

[B57] Proshansky H. M. (1978). The city and self-identity. Environ. Behav. 10, 147–169. 10.1177/0013916578102002

[B58] Ratcliffe E. Korpela K. M. (2016). Memory and place attachment as predictors of imagined restorative perceptions of favourite places. J. Environ. Psychol. 48, 120–130. 10.1016/j.jenvp.2016.09.005

[B59] Robinson J. A. (1986). “Autobiographical memory: a historical prologue,” in Autobiographical Memory, ed. D. C. Rubin (Cambridge: Cambridge University Press). 10.1017/CBO9780511558313.005

[B60] Ru X. Qin H. Wang S. (2019). Young people's behavior intentions towards reducing PM2.5 in China: extending the theory of planned behavior. Resour. Conserv. Recycl. 141, 9–108. 10.1016/j.resconrec.2018.10.019

[B61] Scannell L. Gifford R. (2010a). The relations between natural and civic place attachment and pro-environmental behavior. J. Environ. Psychol. 30, 289–297. 10.1016/j.jenvp.2010.01.010

[B62] Scannell L. Gifford R. (2010b). Defining place attachment: a tripartite organizing framework. J. Environ. Psychol. 30, 1–10. 10.1016/j.jenvp.2009.09.006

[B63] Scannell L. Gifford R. (2013). Personally relevant climate change: the role of place attachment and local versus global message framing in engagement. Environ. Behav. 45, 60–85. 10.1177/0013916511421196

[B64] Shi J. Xu K. Si H. Song L. Duan K. (2021). Investigating intention and behavior towards sorting household waste in Chinese rural and urban-rural integration areas. J. Clean. Prod. 298:126827. 10.1016/j.jclepro.2021.126827

[B65] Shin J. Yang H. J. (2022). Does residential stability lead to civic participation?: the mediating role of place attachment. Cities 126:103700. 10.1016/j.cities.2022.103700

[B66] Si H. Shi J. Tang D. Wu G. Lan J. (2020). Understanding intention and behavior toward sustainable usage of bike sharing by extending the theory of planned behavior. Resour. Conserv. Recycl. 152:104513. 10.1016/j.resconrec.2019.104513

[B67] Stedman R. C. (2003). Toward a social psychology of place. Environ. Behav. 34, 561–581. 10.1177/0013916502034005001

[B68] Sun C. Liu J. Chen Y. (2025). Impact of empowerment on public participation awareness in community renewal projects: mediating role of trust and community identity. J. Asian Architect. Build. Eng. 24, 1931–1944. 10.1080/13467581.2024.2321996

[B69] Tallon A. (2013). Urban regeneration in the UK, 2nd Edn. London: Routledge. 10.4324/9780203802847

[B70] Tang D. Gong X. Liu M. (2022). Residents' behavioral intention to participate in neighborhood micro-renewal based on an extended theory of planned behavior: a case study in Shanghai, China. Habitat Int. 129:102672. 10.1016/j.habitatint.2022.102672

[B71] Taylor S. M. Ward P. Zabriskie R. Hill B. Hanson C. (2012). Influences on active family leisure and a healthy lifestyle among adolescents. Leisure Sci. 34, 332–349. 10.1080/01490400.2012.687643

[B72] Tonglet M. Phillips P. Bates M. (2004). Determining the drivers for householder pro-environmental behavior: waste minimisation compared to recycling. Resour. Conserv. Recycl. 42, 27–48. 10.1016/j.resconrec.2004.02.001

[B73] Tsai C.-T. (2016). Memorable tourist experiences and place attachment when consuming local food. Int. J. Tourism Res. 18, 536–548. 10.1002/jtr.2070

[B74] Tu J.-C. Chen Y.-Y. Lee Y.-L. Wang X.-L. (2020). Investigating the use of environmental tableware based on the theory of planned behavior. Environ. Dev. Sustain. 23, 10013–10037. 10.1007/s10668-020-01044-x

[B75] Von Wirth T. Grêt-Regamey A. Moser C. Stauffacher M. (2016). Exploring the influence of perceived urban change on residents' place attachment. J. Environ. Psychol. 46, 67–82. 10.1016/j.jenvp.2016.03.001

[B76] Wei C. Zhao W. Zhang C. Huang K. (2019). Psychological factors affecting memorable tourism experiences. Asia Pacific J. Tourism Res. 24, 619–632. 10.1080/10941665.2019.1611611

[B77] Westerink J. Kempenaar A. van Lierop M. Groot S. van der Valk A. van den Brink A. (2017). The participating government: shifting boundaries in collaborative spatial planning of urban regions. Environ. Plann. C Polit. Space 35, 147–168. 10.1177/0263774X16646770

[B78] Woosnam K. M. Ribeiro M. A. Denley T. J. Hehir C. Boley B. B. (2022). Psychological antecedents of intentions to participate in last chance tourism: considering complementary theories. J. Travel Res. 61, 1342–1357. 10.1177/00472875211025097

[B79] Wu F. (2016). State dominance in urban redevelopment. Urban Affairs Rev. 52, 631–658. 10.1177/1078087415612930

[B80] Wu R. Li Z. Liu Y. Huang X. Liu Y. (2019). Neighborhood governance in post-reform Urban China: place attachment impact on civic engagement in Guangzhou. Land Use Policy 81, 472–482. 10.1016/j.landusepol.2018.11.019

[B81] Xiao S. Li L. Ma J. Liu D. Li J. (2023). A study of residents' intentions to participate in the renovation of older communities under the perspective of urban renewal: evidence from Zhangjiakou, China. J. Asian Architect. Build. Eng. 22, 1094–1109. 10.1080/13467581.2023.2182643

[B82] Xu X. Xue D. Huang G. (2022). The effects of residents' sense of place on their willingness to support urban renewal: a case study of century-old East Street Renewal project in Shaoguan, China. Sustainability 14:1385. 10.3390/su14031385

[B83] Xu Z. Shan J. Li J. Zhang W. (2020). Extending the theory of planned behavior to predict public participation behavior in air pollution control: Beijing, China. J. Environ. Plann. Manage. 63, 669–688. 10.1080/09640568.2019.1603821

[B84] Xu Z. Zhang J. Geoffrey W. Cao J. Zhang H. (2009). Research on influence of residents' place attachment on positive attitude to tourism with a mediator of development expectation: a case of core tourism community in Jiuzhaigou. Acta Geogr. Sin. 64, 736–744.

[B85] Zhai B. Ng M. K. (2013). Urban regeneration and social capital in China: a case study of the Drum Tower Muslim District in Xi'an. Cities 35, 14–25. 10.1016/j.cities.2013.05.003

[B86] Zhang H. Cong C. Chakraborty A. (2022). Exploring the institutional dilemma and governance transformation in China's urban regeneration: based on the case of Shanghai Old Town. Cities 131:103915. 10.1016/j.cities.2022.103915

[B87] Zhang H. Wu Y. Buhalis D. (2018). A model of perceived image, memorable tourism experiences and revisiting intention. J. Destination Market. Manage. 8, 326–336. 10.1016/j.jdmm.2017.06.004

[B88] Zhang J. Quoquab F. Mohammad J. (2024b). Metaverse tourism and Gen-Z and Gen-Y's motivation: “will you, or won't you travel virtually?” Tourism Rev. 79, 304–320. 10.1108/TR-06-2023-0393

[B89] Zhang Z. Yu J. Tian J. (2024a). Community participation, social capital cultivation and sustainable community renewal: a case study from Xi'an's Southern Suburbs, China. J. Knowl. Econ. 15, 11007–11040 10.1007/s13132-023-01536-x

[B90] Zhou J. Yan S. Wan Z. (2019). Reflections on improving the urban renewal system of Shanghai. Urban Plann. Forum 1, 20–26. 10.16361/j.upf.201901002

[B91] Zhu Y. (2022). Interests driven or socially mobilized? Place attachment, social capital, and neighborhood participation in urban China. J. Urban Aff. 44, 1136–1153. 10.1080/07352166.2020.1773837

